# AMH predicts miscarriage in non-PCOS but not in PCOS related infertility ART cycles

**DOI:** 10.1186/s12958-023-01087-5

**Published:** 2023-04-05

**Authors:** Christopher Arkfeld, Eric Han, Reshef Tal, David B. Seifer

**Affiliations:** 1 Division of Reproductive Endocrinology and Infertility, Department of Obstetrics, Gynecology, and Reproductive Sciences, Yale University School of Medicine, New Haven, CT USA; 2 Yale New Haven Hospital, 20 York Street, New Haven, CT 06510 USA

**Keywords:** AMH, Miscarriage, PCOS, IVF

## Abstract

**Background:**

To study whether AMH levels were associated with miscarriage rates in index ART cycles undergoing fresh autologous transfers in PCOS and non-PCOS related infertility.

**Methods:**

In the SART CORS database 66,793 index cycles underwent fresh autologous embryo transfers with AMH values reported within the last 1-year between 2014 and 2016. Cycles that resulted in ectopic or heterotopic pregnancies, or were performed for embryo/oocyte banking were excluded.

Data were analyzed using Graphpad Prism-9. Odds ratios (OR) were calculated with 95% confidence intervals (CI) along with multivariate regression analysis adjusting for age, body mass index (BMI), and number of embryos transferred. Miscarriage rates were calculated as miscarriage per clinical pregnancies.

**Results:**

Of the total 66,793 cycles, the mean AMH was 3.2 ng/ml and were not associated with increased miscarriage rates for AMH < 1 ng/ml (OR 1.1, CI 0.9–1.4, *p =* 0.3). Of the 8,490 PCOS patients, the mean AMH was 6.1 ng/ml and were not associated with increased miscarriage rates for AMH < 1 ng/ml (OR 0.8, CI 0.5–1.1, *p =* 0.2). Of the 58,303 non-PCOS patients, the mean AMH was 2.8 ng/ml and there was a significant difference in miscarriage rates for AMH < 1 ng/ml (OR 1.2, CI 1.1–1.3, *p <* 0.01). All findings were independent of age, BMI and number of embryos transferred. This statistical significance did not persist at higher thresholds of AMH. The overall miscarriage rate for all cycles, and cycles with and without PCOS were each 16%.

**Discussion:**

The clinical utility of AMH continues to increase as more studies investigate its predictive abilities regarding reproductive outcomes. This study adds clarity to the mixed findings of prior studies that have examined the relationship between AMH and miscarriage in ART cycles.

AMH values of the PCOS population are higher than the non-PCOS. The elevated AMH associated with PCOS decreases its utility in predicting miscarriages in IVF cycles as it may be representing the number of developing follicles rather than oocyte quality in the PCOS patient population. The elevated AMH associated with PCOS may have skewed the data; removing this sub-population may have unmasked significance within the non-PCOS associated infertility.

**Conclusions:**

AMH < 1 ng/mL is an independent predictor of increased miscarriage rate in patients with non-PCOS infertility.

**Supplementary Information:**

The online version contains supplementary material available at 10.1186/s12958-023-01087-5.

## Introduction

Anti-mullerian hormone (AMH) is a member of the transforming growth factor β (TGF-β) expressed by granulosa cells of preantral and small antral follicles within the ovary [1]. Serum AMH levels rise from birth and plateau around 25 years of age, after which it is inversely associated with advancing age [2, 3]. Overall, AMH has become a well-established marker of functional ovarian reserve [4–6]. Ovarian stimulation has a known dose dependent response to AMH levels and elevated levels, as present in polycystic ovarian syndrome (PCOS), are associated with increased risk of ovarian hyperstimulation syndrome (OHSS) [7]. AMH has also been shown to have weak predictive ability for pregnancy and live birth [8, 9]. However, the utility of AMH in predicting miscarriage in assisted reproductive technology (ART) cycles is unresolved with some studies concluding that increased AMH levels were associated with decreased miscarriage rates [10]. This inconsistency may be due to the inclusion of patients with PCOS related infertility in prior studies as the association of PCOS with abnormally elevated AMH may mask an association between low AMH levels and the probability of miscarriage in non-PCOS women with infertility [11–13].

Our objective was to examine the Society for Assisted Reproductive Technology Clinic Outcome Reporting System (SART CORS) database to examine whether AMH levels were associated with miscarriage rates in initial index IVF/ICSI cycles undergoing fresh autologous transfers in non-PCOS and PCOS related infertility.

## Materials and methods

The data used for this study were obtained from the SART CORS. Data were collected through voluntary submission, verified by SART, and reported to the Centers for Disease Control and Prevention (CDC) in compliance with the Fertility Clinic Success Rate and Certification Act of 1992 (Public Law 102–493). SART maintains HIPAA-compliant business associates agreements with reporting clinics. In 2004, following a contract change with the CDC, SART gained access to the SART CORS data system for the purposes of conducting research. In 2019, 81% of clinics were SART members reporting 90% of all IVF cycles in the United States [14].

The data in the SART CORS are validated annually with some clinics receiving on-site visits for chart review based on an algorithm for clinic selection. During each visit, data reported by the clinic were compared with information recorded in patients’ charts. In 2021, records for 1,945 cycles at 33 clinics were randomly selected for full validation, along with 262 fertility preservation cycles selected for partial validation. Nine out of ten data fields selected for validation were found to have discrepancy rates of ≤ 5% [14]. The exception was the diagnosis field, which, depending on the diagnosis, had a discrepancy rate between 0.7% and 9.1%.

For this study, the SART CORS database identified 533,463 cycles reported between 2014 and 2016. Only initial index cycles of patients who underwent fresh autologous embryo transfers with AMH values reported within the last 1 year were included. Index cycles were defined as the initial cycle for each patient identified documented in the SART CORS database. Thus, each cycle represented a single patient and there were no patients with multiple cycles included in this study. Cycles that underwent preimplantation genetic testing, resulted in ectopic or heterotopic pregnancies, that were performed for embryo or oocyte banking, or cycles performed for a transfer to a gestational carrier were excluded, see Appendix Figure 2 for summary of excluded cycles. For the included 66,793 cycles, we examined cycles with AMH values of < 1 ng/ml. We chose AMH < 1 ng/ml as a widely accepted threshold as a low age-specific AMH value in women ≤ 40 years old.

Data were analyzed using Graphpad Prism-9. Odds ratios of miscarriage for AMH < 1 ng/ml were calculated with 95% confidence intervals (CI). Multivariate regression analysis was performed adjusting for age, body mass index (BMI), and number of embryos transferred.

Secondary analysis was performed on non-PCOS (58,303 cycles) and PCOS (8,490 cycles) patients separately given the association between PCOS and elevated AMH. The diagnosis of PCOS was identified as a designated category in the SART CORS database. Miscarriage rates were calculated as number of miscarriages per clinical pregnancies. Miscarriage was determined by documented intrauterine pregnancies (IUPs) with cardiac activity that did not result in a live or still birth.

This study was approved by the SART research committee and was exempted from review by the institutional review board of Yale School of Medicine.

## Results

The baseline characteristics of the non-PCOS infertility patients were notable for having a lower mean AMH of 2.8 ng/ml compared to 6.1 ng/ml in the patients with PCOS. The baseline characteristics of non-PCOS and PCOS patients are summarized in Table 1. The etiology of infertility was documented according to the type of infertility field in the SART CORS database. The etiologies of infertility included in the analysis were diminished ovarian reserve, male factor, tubal factor, endometriosis, uterine factor, PCOS related infertility, and unexplained infertility. The patient distribution of each etiology is summarized in Table 1.

**Table 1:** Summary of baseline characteristics

Baseline Characteristics	Non-PCOS (*n=*58,303)	PCOS (*n=*8,490)	Total Cycles (*n=* 66,793)	*P* value
	Average +/- SD (Range)	Average +/- SD (Range)	Average +/- SD (Range)	
Age	34.6 +/- 4.5 (17-52)	32.4 +/- 4.1 (19-46)	34.3 +/- 4.5 (17-52)	<0.01
AMH (ng/mL)	2.8 +/- 2.6 (0-66)	6.1 +/5.2 (0-68.2)	3.2 +/- 3.3 (0-68.2)	<0.01
BMI (Kg/m2)	26 +/- 6 (10.7-161.2)	28.1 +/- 7.2 (10.65-161.2)	26.3 +/- 6.2 (10.7-161.2)	<0.01
Gravidity	0.9 +/- 1.3 (0-10)	0.7 +/-1.1 (0-10)	0.9 +/- 1.3 (0-10)	<0.01
% Non-Caucasian	31923 (55%)	4148 (49%)	30071 (54%)	<0.01
**Infertility Etiology**				
	*n* (%)	*n* (%)	*n* (%)	
Diminished Ovarian Reserve	15165 (26%)	337 (4%)	15502 (23%)	<0.01
Male Factor	23304 (40%)	2702 (31%)	26006 (39%)	<0.01
Tubal	9870 (17%)	620 (7.3%)	10556 (16%)	<0.01
Endometriosis	5769 (9.9%)	493 (5.8%)	6262 (9.4%)	<0.01
Unexplained	10525 (18%)	0 (0%)	10525 (16%)	Not calculated
Uterine	2783 (4.8%)	322 (3.8%)	3105 (4.6%)	<0.01
PCOS	0 (0%)	8490 (100%)	8490 (13%)	Not Calculated

*AMH* Anti-mullerian hormone, *BMI* Body mass index, *PCOS* Polycystic ovarian syndrome, *SD* Standard deviation

Cycle specific data was also collected. Specifically, the number of oocytes retrieved per cycle and the number of embryos transferred per cycle were analyzed. The summary of stimulation responses including number of oocytes retrieved and embryos transferred are summarized in Table 2.

**Table 2:** Summary of stimulation results

**Stimulation Characteristics**	**Non-PCOS (** ***n=*** **58,303)**	**PCOS (** ***n=*** **8,490)**	**Total Cycles (** ***n=*** ** 66,793)**	***P*** ** value**
	**Average +/- SD (Range)**	**Average +/- SD (Range)**	**Average +/- SD (Range)**	
No. Eggs Retrieved	12.1 +/- 7.3 (1-99)	16 +/- 8.6 (1-92)	12.6 +/- 7.6 (1-99)	<0.01
No. Embryos Transferred	1.8 +/- 0.7 (1-10)	1.7 +/- 0.6 (1-8)	1.8 +/- 0.73 (1-10)	<0.01

*PCOS* Polycystic ovarian syndrome, *SD* Standard deviation

In the combined analysis of all patients with infertility which included 66,793 cycles (non-PCOS and PCOS cycles), patient’s mean age was 34.3 years with a mean BMI of 26.3 kg/m^2^. The mean AMH was 3.2 ng/ml, and the mean number of embryos transferred was 1.8. AMH levels were not associated with increased miscarriage rates for AMH < 1 ng/ml (OR 1.1, CI 0.9–1.4, *p =* 0.3) independent of age, BMI and number of embryos transferred when examining all patient cycles regardless of etiology of infertility.

Of the non-PCOS patients alone which included 58,303 cycles, the mean age was 34.6 years with a mean BMI of 26 kg/m^2^. The mean AMH was 2.8 ng/ml, and the mean number of embryos transferred was 1.8. In non-PCOS cycles, there was a significant difference in miscarriage rates for AMH < 1 ng/ml (OR 1.2, CI 1.1–1.3, *p <* 0.01) independent of age, BMI and number of embryos transferred, unadjusted means are summarized in Table 3. This statistical significance did not persist at higher thresholds of AMH ≥ 1 ng/ml, see Appendix Supplementary Table 1. The overall miscarriage rate for all index cycles, and cycles with or without PCOS was 16%.

**Table 3:** Unadjusted baseline characteristics for non-PCOS patients

	**Total Count (cycles)**	**Age (years)**	**BMI (kg/m2)**	**Gravidity**	**No. Eggs Retrieved**	**No. Embryos Transferred**
**AMH <1**	13896	36.8, (19-52)	26.3, (10.7-64.6)	1, (0-10)	6, (1-55)	1.9, (1-8)
**AMH ≥1**	52901	33.6, (17-50)	26.2, (10.7-73.2)	0.8, (0-10)	14.2, (1-93)	1.7, (1-10)

*AMH* Anti-mullerian hormone, *BMI* Body mass index

Of the PCOS patients alone, which included 8,490 cycles, the mean age was 32.4 years with a mean BMI of 28.1 kg/m [2]. The mean AMH was 6.1 ng/ml, and the mean number of embryos transferred was 1.7. AMH levels were not associated with increased miscarriage rates for AMH < 1 ng/ml (OR 0.8, CI 0.5–1.1, *p =* 0.2) independent of age, BMI and number of embryos transferred.

**Fig. 1:**
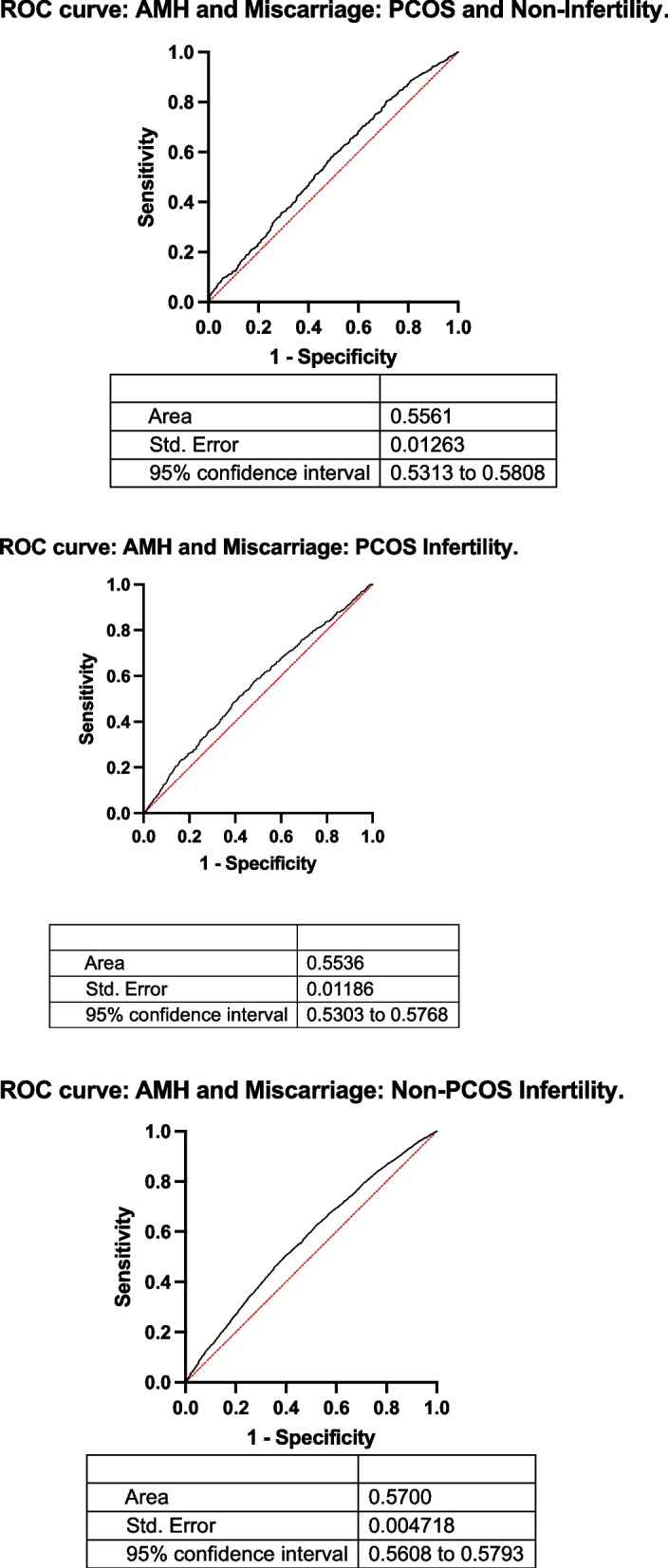
ROC curve for all subjects, all patients with PCOS related infertility, and non-PCOS related infertility. Legend: AMH *anti-mullerian hormone*, PCOS *polycystic ovarian syndrome*

When analyzed as a continuous variable AMH was a statistically significant predictor of miscarriage for non-PCOS patients (OR 0.97, CI 0.96–0.98). Receiver operating characteristic (ROC) curves were performed, the area under the curve (AUC) were calculated with 95% confidence interval (CI). The 95% confidence interval for AUC for non-PCOS patients was 0.56–0.58 and PCOS patients was 0.53–0.58. Thus, the ROC analysis suggested that AMH was a weak independent predictor of miscarriage after ART (Appendix: Figure 1).

## Discussion

These data show an association between low serum AMH values in women with non-PCOS related infertility and miscarriage following ART. This study adds clarity to the mixed findings of prior studies that have examined the relationship between AMH and miscarriage in ART cycles. Since this study analyzed initial index cycles that underwent fresh transfers, none of the cycles underwent pre-implantation genetic testing. This study improves our ability to provide patient counseling before the start of their initial cycle.

It has been previously shown that the AMH values of the PCOS population are higher than the non-PCOS population [12] and that the higher the AMH, the greater the severity of PCOS [13]. The abnormally elevated AMH levels associated with PCOS likely decrease its utility in predicting miscarriages in IVF cycles as it correlates with the high AMH-secreting arrested follicles rather than oocyte quality in the PCOS patient population [15]. We speculate that the elevated AMH associated with PCOS appears to skew the data when examining all patients with infertility. Thus, removing this sub-population (ie. PCOS) may have unmasked the association of miscarriage in women with AMH < 1 ng/ml with non-PCOS associated infertility.

Existing literature highlight the inconsistent findings regarding AMH and miscarriage rates. Some studies that have found no association between AMH and miscarriage rates included PCOS related infertility in their study population [16]. Other studies attempted to remove PCOS related infertility patients based on AMH levels > 6 ng/ml, which likely did not adequately exclude all PCOS patients [17]. Prior studies have reviewed AMH’s ability to predict live birth and miscarriage and have found it is inversely associated with miscarriage in naturally conceived pregnancies [10, 18]. Similarly, AMH has been strongly associated with cumulative live birth rate in women with diminished ovarian reserve [19]. We believe these inconsistent findings may be related to inclusion of cycles for both PCOS and non-PCOS related infertility. Some studies have noted similar findings, low AMH of 0.08–1.6 ng/ml, being associated with miscarriage independent of age in IVF cycles [20]. AMH is correlated with clinical live birth rate in patients with diminished ovarian reserve regardless of their age [19]. The present study examined a broader patient group including other types of infertility beyond diminished ovarian reserve.

Low AMH has been previously associated with recurrent pregnancy loss (RPL) [21]. This finding persisted even after excluding women with PCOS. One limitation of this study is that RPL was not listed in the SART CORS database as a specific data field and thus we were unable to control for RPL as a confounder. However, given that the estimated prevalence of RPL within the population is 1–2%,we do not anticipate inclusion of RPL would have affected our reported findings [22]. Noting that higher AMH was associated with increased miscarriage rates within the PCOS related infertility patient population suggests that different levels of AMH may be representing different ongoing biomedical processes related to sustainable implantation within PCOS and non-PCOS patients [23]. This is an area for further investigation.

Additional research may also be warranted to better identify the most clinically relevant cutoff for AMH values. Previous studies have used 0.4 ng/ml when looking at naturally conceived pregnancies [10]. Our study supports the clinical utility of a higher cutoff of < 1 ng/ml when counseling the non-PCOS patient undergoing ART on the risk of miscarriage before undergoing their initial ART cycle.

## Conclusions

AMH of < 1 ng/mL is an independent predictor of increased miscarriage rate in patients with non-PCOS infertility undergoing ART without pre-implantation genetic testing.

During initial index cycles of all combined patients with a diagnosis of PCOS and non-PCOS infertility undergoing fresh autologous embryo transfers, AMH < 1 ng/ml was not associated with increased miscarriage rates. Given these findings, AMH is of limited clinical value for predicting miscarriage in cycles with PCOS but may offer clinical utility for women with non-PCOS related infertility patients undergoing ART. Both AMH and age-based counseling should be considered when discussing the probability of clinical success for women without PCOS related infertility when undergoing ART.

### Supplementary Information

**Additional file 1: Supplementary Table 1.** Summary of AMH Strata of Non-PCOS patients’ association with Miscarriage

## Figures and Tables

**Fig. 2:**
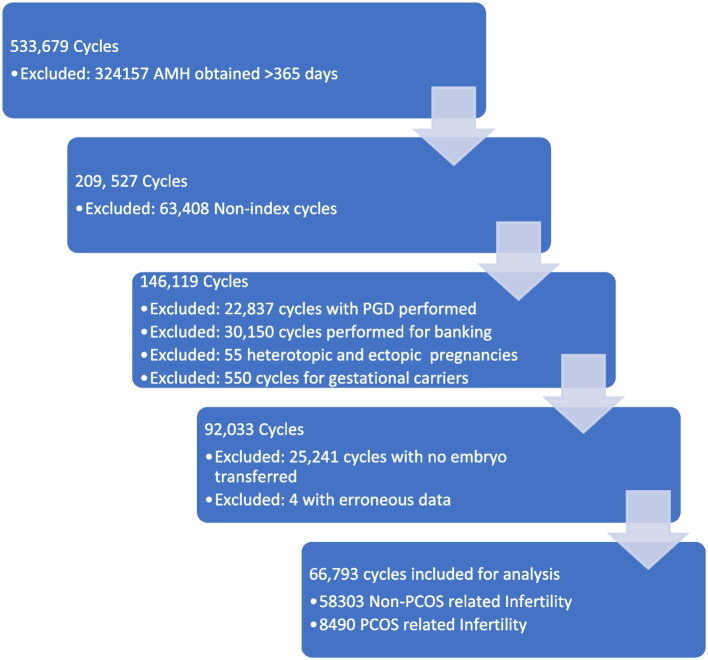
Flow chart summarizing cycles excluded from analysis. Legend: AMH *anti-mullerian hormone*, PGD *preimplantation genetic testing*, PCOS *polycystic ovarian syndrome*

## Data Availability

The data that support the findings of this study are available from SART CORS but restrictions apply to the availability of these data, which were used under license for the current study, and so are not publicly available. Data are however available from the authors upon reasonable request and with permission of SART CORS.

## Abbreviations

AMH: Anti mullerian hormone
PCOS: Polycystic ovarian syndrome
IVF: In vitro fertilization
BMI: Body mass index
ICSI: Intracytoplasmic sperm injection
SART CORS: Society for assisted Reproductive Technology Clinic Outcome Reporting System

## References

[1] di Clemente N, Racine C, Pierre A, Taieb J (2021). Anti-Mullerian Hormone in Female Reproduction. Endocr Rev.

[2] Kelsey TW, Wright P, Nelson SM, Anderson RA, Wallace WH (2011). A validated model of serum anti-mullerian hormone from conception to menopause. PLoS ONE.

[3] Lie Fong S, Visser JA, Welt CK (2012). Serum anti-mullerian hormone levels in healthy females: a nomogram ranging from infancy to adulthood. J Clin Endocrinol Metab.

[4] Fleming R, Seifer DB, Frattarelli JL, Ruman J (2015). Assessing ovarian response: antral follicle count versus anti-Mullerian hormone. Reprod Biomed Online.

[5] Seifer DB, MacLaughlin DT, Christian BP, Feng B, Shelden RM (2002). Early follicular serum mullerian-inhibiting substance levels are associated with ovarian response during assisted reproductive technology cycles. Fertil Steril.

[6] Tal R, Seifer DB (2017). Ovarian reserve testing: a user's guide. Am J Obstet Gynecol.

[7] Vembu R, Reddy NS (2017). Serum AMH Level to Predict the Hyper Response in Women with PCOS and Non-PCOS Undergoing Controlled Ovarian Stimulation in ART. J Hum Reprod Sci.

[8] Tal R, Seifer DB, Wantman E, Baker V, Tal O (2018). Antimullerian hormone as a predictor of live birth following assisted reproduction: an analysis of 85,062 fresh and thawed cycles from the Society for Assisted Reproductive Technology Clinic Outcome Reporting System database for 2012–2013. Fertil Steril.

[9] Iliodromiti S, Anderson RA, Nelson SM (2015). Technical and performance characteristics of anti-Mullerian hormone and antral follicle count as biomarkers of ovarian response. Hum Reprod Update.

[10] Lyttle Schumacher BM, Jukic AMZ, Steiner AZ (2018). Antimullerian hormone as a risk factor for miscarriage in naturally conceived pregnancies. Fertil Steril.

[11] Cornille AS, Sapet C, Reignier A, et al. Is low anti-Mullerian hormone (AMH) level a risk factor of miscarriage in women <37 years old undergoing in vitro fertilization (IVF)? Hum Fertil (Camb) 2021:1–11. 10.1080/14647273.2021.1873431. 10.1080/14647273.2021.1873431 33448232

[12] Hart R, Doherty DA, Norman RJ (2010). Serum antimullerian hormone (AMH) levels are elevated in adolescent girls with polycystic ovaries and the polycystic ovarian syndrome (PCOS). Fertil Steril.

[13] Tal R, Seifer DB, Khanimov M, Malter HE, Grazi RV, Leader B (2014). Characterization of women with elevated antimullerian hormone levels (AMH): correlation of AMH with polycystic ovarian syndrome phenotypes and assisted reproductive technology outcomes. Am J Obstet Gynecol.

[14] CDC. Centers for Disease Control and Prevention. 2019 Assisted Reproductive Technology Fertility Clinic and National Summary Report. 2021.

[15] Dumont A, Robin G, Catteau-Jonard S, Dewailly D (2015). Role of Anti-Mullerian Hormone in pathophysiology, diagnosis and treatment of Polycystic Ovary Syndrome: a review. Reprod Biol Endocrinol.

[16] Li XL, Huang R, Fang C, Liang XY (2018). Basal Serum Anti-Mullerian Hormone Level as a Predictor of Clinical Outcomes in Freezing-all Embryo Transfer Program. Curr Med Sci.

[17] Peuranpaa P, Hautamaki H, Halttunen-Nieminen M, Hyden-Granskog C, Tiitinen A (2020). Low anti-Mullerian hormone level is not a risk factor for early pregnancy loss in IVF/ICSI treatment. Hum Reprod.

[18] Busnelli A, Somigliana E, Cirillo F, Levi-Setti PE (2021). Is diminished ovarian reserve a risk factor for miscarriage? Results of a systematic review and meta-analysis. Hum Reprod Update.

[19] Tal R, Seifer DB, Tal R, Granger E, Wantman E, Tal O (2021). AMH Highly Correlates With Cumulative Live Birth Rate in Women with Diminished Ovarian Reserve Independent of Age. J Clin Endocrinol Metab.

[20] Tarasconi B, Tadros T, Ayoubi JM, Belloc S, de Ziegler D, Fanchin R (2017). Serum antimullerian hormone levels are independently related to miscarriage rates after in vitro fertilization-embryo transfer. Fertil Steril.

[21] Leclercq E, de Saint ML, Bohec C (2019). Blood anti-Mullerian hormone is a possible determinant of recurrent early miscarriage, yet not conclusive in predicting a further miscarriage. Reprod Biomed Online.

[22] Ford HB, Schust DJ (2009). Recurrent pregnancy loss: etiology, diagnosis, and therapy. Rev Obstet Gynecol.

[23] Mayrhofer D, Hager M, Walch K (2020). The Prevalence and Impact of Polycystic Ovary Syndrome in Recurrent Miscarriage: A Retrospective Cohort Study and Meta-Analysis. J Clin Med.

