# Supplementary material for: AMH predicts miscarriage in non-PCOS but not in PCOS related infertility ART cycles
Source: Reprod Biol Endocrinol. 2023 Apr 5;21:35. doi: 10.1186/s12958-023-01087-5 (PMC10074664; doi:10.1186/s12958-023-01087-5)
Supplement: Supplementary file 1 — Additional file 1: Supplementary Table 1. Summary of AMH Strata of Non-PCOS patients’ association with Miscarriage [file 12958_2023_1087_MOESM1_ESM.docx]

**Supplementary Table 1: Summary of AMH Strata of Non-PCOS patients’ association with Miscarriage**

|  | **AMH <1** **ng/ml** | **P Value** | **AMH 1-5** **ng/ml** | **P Value** | **AMH 5-10** **ng/ml** | **P Value** |
| --- | --- | --- | --- | --- | --- | --- |
|  | **OR (CI)** |  | **OR (CI)** |  | **OR (CI)** |  |
| Non-PCOS patients | 1.2 (1.1-1.3) | <0.01 | 0.8 (0.7-0.9) | <0.01 | 0.8 (0.7-1.1) | 0.1 |
| PCOS patients | 0.8 (0.5-1.1) | 0.2 | 1.4 (1.2-1.6) | <0.01 | 1.4 (1.1-1.7) | <0.01 |

Legend: AMH *anti-mullerian hormone*, PCOS *polycystic ovarian syndrome*
